# Qualitative digital diary methods: participant-led values for ethical and insightful mental health research

**DOI:** 10.1177/16094069241296189

**Published:** 2024-11-26

**Authors:** Catherine McCombie, Georgina Miguel Esponda, Hannah Ouazzane, Gemma Knowles, Charlotte Gayer-Anderson, Ulrike Schmidt, Vanessa Lawrence

**Affiliations:** 1 Department of Health Services and Population Research, Institute of Psychiatry, Psychology & Neuroscience, King’s College London, London, UK; 2 ESRC Centre for Society and Mental Health, King’s College London, UK; 3 Section of Eating Disorders, Department of Psychological Medicine, Institute of Psychiatry, Psychology & Neuroscience, King’s College London, London, UK; 4 South London and Maudsley NHS Foundation Trust, London, UK

**Keywords:** qualitative diary methods, digital research, participant perspectives, research ethics

## Abstract

Qualitative digital diary methods are a promising tool for capturing participants’ experiences in their own words and over time. The use of smartphone apps to collect this kind of data provides an accessible and flexible way to participate in research, but to truly benefit from this method, participants needs and preferences must be taken into account. This paper explores participants’ experiences of taking part in qualitative digital diary research, and highlights participants’ values and priorities for qualitative digital diary mental health research. Participants from two qualitative digital diary studies provided feedback on their experiences, in the form of interviews and focus groups, and data were analysed using reflexive thematic analysis. The two participant groups, people with lived experience of eating disorders, and young people from diverse backgrounds across London schools, allowed exploration of experiences across different contexts and populations. The six resulting themes each reflect a core value that participants identified as an essential component for them in qualitative digital diary research: Self-expression, flexibility, non-judgement, open communication, helpful reflection, and meaningful impact. Themes each highlight aspects of participants’ experiences that must be taken into account for future research to ensure that participants can take part in this type of research in ways that are meaningful to them, as well as most beneficial to the research. This paper provides an overview of participant experiences of qualitative digital diary research, and provides a framework for centring participant values and preferences in future qualitative diary research.

## Introduction

Qualitative research practices are expanding in the digital age, yet the potential of qualitative digital diaries remains under-explored by the mental health research community. Qualitative digital diary methods (QDMs) offer a way for participants to record their thoughts, feelings, and interpretations in their own way and across a period of time, providing detailed insights into phenomena within the context of their lives (Craig et al., 2017). The increasing availability of mobile phone applications for research provides a uniquely accessible way for participants to record their experiences. While this can increase research accessibility and involvement, it also poses ethical challenges that require careful consideration. This paper provides findings from a study exploring participant experiences of taking part in qualitative digital diary research. We firstly highlight the value of qualitative digital diary methods, and stress the importance of prioritising participant preferences in this type of research, then outline the methods and participants. The findings are then presented, in the form of six core participant values for taking part in qualitative digital diary research. Finally, implications from this for future research are considered.

Qualitative diaries can provide insight into a broader range of thoughts and feelings than is typically present in interviews, and allow an unfolding narrative to develop throughout diary entries, in contrast to the more singular line of thinking present in interviews at one time point, which may also be influenced by the presence of the researcher (Herron et al., 2019). Additionally, the nature of diaries and how they are recorded over time means that the diarist is recording meanings as they interpret and reinterpret them over time, giving insight into participants’ meaning-making, and evaluations of their experiences (Day & Thatcher, 2009). Greenaway et al. (2018) highlight that context influences the expression of emotion, and that all narratives are co-created between the speaker and the audience (Andrews et al., 2008). Diaries, with the audience more removed than in an interview or focus group study, may provide access to a different kind of narrative that is more directed by the participant, less socially constrained, and using more free and expressive language. Diaries also mean that participants can record what they want, what is important to them, and their own reflections on this, thus putting participants more in control of data collection than in other methods (Bijoux & Myers, 2006). Several studies have demonstrated the benefits of using qualitative diary methods in mental health research, and our scoping review of this highlights benefits and challenges of this approach (McCombie, Miguel Esponda, et al., 2023). QDMs can therefore offer a way for participants to take part in research in a more natural and reflective way for them, and in a way that provides further insight and understanding into the experience and contexts of mental health difficulties.

However, QDMs are a hugely diverse method, with many design options and iterations, and it is possible for diaries to be intrusive, burdensome, and difficult for participants to keep in meaningful ways. Intrusiveness and burden are key issues particularly in digital diary research, as the ubiquitous nature of smartphones means participants could be considered to be constantly available to provide research data. To support participant engagement in this research, researchers need to understand participant preferences for using digitial diaries (Mendoza et al., 2021). Qualitative digital diary methods, and indeed any research method, are only valuable and ethical if they prioritise and reflect participants’ needs and values. This is particularly important when conducting research with people with mental health difficulties, where enabling participants to communicate their experiences in ways that make sense to them is vital due to risks of misinterpretations or misunderstanding by researchers or clinicians, and the general epistemic injustice that mental health patients face (Crichton et al., 2017).

It is therefore important to understand how individuals with experience of mental health difficulties or contexts that can give rise to distress would like to take part in qualitative digital diary research. The objective of this study was to understand the perspectives of two different participant groups on their experiences of taking part in qualitative digital diary research, and to explore what can be learned from this in terms of participants’ core values for qualitative digital diary research.

## Methods

### Study outline

This paper uses data from two qualitative digital diary studies - the Eating Disorder Recovery Diary Study, and the REACH Diary Study, and hence two different groups of participants: young people in London schools (the REACH diary study) and adults recovering from an eating disorder (the Eating Disorder Recovery Diary Study). These two participant groups allowed exploration of experiences of qualitative digital diary research across different contexts and populations, supporting the findings to reflect key aspects of experiences across the different groups. Paticipants in each study kept a qualitative digital diary, and then took part in follow-up stages to discuss their experiences of taking part in the QDM research. A total of 49 participants across both studies took part in these follow-up stages. The findings from the diary phases of these studies are reported elsewhere (Lawrence et al., Under review; McCombie, Ouzanne, et al., 2023). The current paper focuses on the second stage of the research - exploring participants’ experiences of taking part.

### Eating Disorder Recovery Diary Study

This study entailed adult participants keeping diaries documenting their experiences of recovery from eating disorders. Diary data collection took place over November 2021 - March 2022, using a mobile phone app designed for research, Expiwell (2023). Participants kept diaries reflecting on their experiences of maintaining recovery from an eating disorder on that day. Following a two-week diary-keeping period, during which participants submitted diaries every other day, participants were invited to take part in a focus group to discuss their experiences of taking part in the study, and perspectives and preferences for qualitative diary research in the future. The app notified participants each time a diary entry was due, with a subsequent reminder if it was not completed. Short prompts included with the notification suggested recovery-focused questions to consider for each diary entry, and guidance provided at the outset highlighted that they should not spend more than 5-10 minutes on each diary entry, using text writing or audio recording. There was no contact between the researchers and participants during the diary-keeping period, other than to manage technical problems for two participants, but participants were aware they could contact the researchers for support if necessary during the study, for example, if they felt that taking part in the study was negatively affecting their mental health.

### REACH Diary Study

The second data set is from a diary study nested within an ongoing cohort study of adolescent mental health in inner-city London, REACH (Resilience, Ethnicity, and Adolescent Mental Health; Knowles et al., 2022). REACH began in 2016/17 and is investigating the extent, nature, and development of mental health problems among young people from diverse backgrounds. The purpose of the nested Diary Study was to explore young people’s experiences of school closures, lockdowns, and social restrictions during the covid-19 pandemic, with a particular focus on young people from disadvantaged and marginalised backgrounds. Diary data collection took place from October 2020 to March 2021, where participants kept weekly diaries, via a mobile phone app designed for research (MetricWire), over an eight week period, detailing their experiences and the impact of these on their wellbeing. The app sent participants a notification once a week, containing short prompts suggesting topics for the diary entries, with guidance provided at the outset of the study suggesting spending no more than 5-10 minutes on each diary entry using either video, text, or audio recording. Researchers monitored diary entries weekly, and if any indicators of distress were contained within the diary would contact the participants to check in and provide support if necessary. Participants took part in an interview at the start and end of the diary keeping period. In the final interview, participants were invited to discuss including their experiences of taking part in a qualitative diary study.

### Participants

#### Eating Disorder Recovery Diary Study

Participants were recruited online, and needed to self-identify as recovered from a formally diagnosed eating disorder and be currently free from clinically severe levels of eating disorder symptoms (assessed using the Eating Disorder Examination Questionnaire; Fairburn & Beglin, 2008). Fourteen participants kept diaries, and all were interested in taking part in focus groups following the diary period. Due to scheduling difficulties, nine participants ultimately took part in online focus groups. Participants were not offered compensation to take part. Participant characteristics are described in Table 1 below.

#### REACH Diary Study

Participants for the Diary Study were recruited through purposive sampling of REACH participants to ensure a balance of genders, ethnic groups, household income (based on free school meals status), and those scoring high (>18) on the Strengths and Difficulties Questionnaire, a widely used self-report measure of emotional and behavioural difficulties (Goodman, 2001). A study invitation was sent out to 160 REACH participants. Of these 62 responded and 48 were selected to take part. All were offered the option to take part in a interview following the diary keeping period, and forty participants took part in this interview. Participants were offered shopping vouchers of value based on the number of diary entries completed, and additional compensation for taking part in the interview. Participant characteristics can be found in Table 2 below.

### Data collection

#### Eating Disorder Recovery Diary Study

Three online focus groups were held, each involving three participants and two facilitators (CM and HO). Focus groups were considered a suitable forum for participants to discuss and compare their experiences of taking part in the diary study, allowing participants to respond to points raised by others and compare their experiences. As the study population consisted of adults who had recovered from an eating disorder, and the study aim was to find about their experiences of the study rather than their recovery experiences, we felt that a focus group would be an appropriate format with which participants would be comfortable.

A topic guide was used to shape the discussion, although this was used flexibly to allow participants to guide discussions and focus attention on issues important to them. Interviews were held online in Microsoft Teams, and ground rules and general technical guidelines were discussed at the outset. Following agreement from all participants, recording was started and the focus group began. Participants were encouraged to use the online environment as best suited them, including using the chat to talk, and feeling no obligation to turn on their camera if they did not feel comfortable. However, all participants except one used their cameras for the duration, and all participants spoke freely using their microphones rather than writing questions or discussion points in the chat box, which enabled the discussion to flow freely and naturally. Focus groups were around 90 minutes each. Mindful of the strain of being present in online spaces with cameras on, we offered participants a break at around 50 minutes; all chose to continue rather than have a break. The focus groups were audio recorded and transcribed verbatim. All participants provided written informed consent before taking part in the study, which was approved by the King’s College London Health Faculties Research Ethics Subcommittee, reference HR/DP-21/22-26071.

### REACH Diary Study

Participants took part in online interviews, which were transcribed verbatim. Interviews were chosen for this study as the primary focus of the interview was to explore participants’ experiences and their mental health and wellbeing during the Covid-19 pandemic, and it was felt that individual interviews with a researcher would be the most appropriate way to support participants to talk freely about their experiences. A topic guide was used to support the interviews, and covered questions around their lives and experiences during the diary period, and specific questions about experiences of taking part in the dairy study itself.

For the current study, only the parts of the interviews relating directly to experiences of taking part in the diary study were used for analysis. All participants provided written informed consent before taking part in the study, and the study was approved by the King’s College London Psychiatry, Nursing and Midwifery Research Ethics Subcommittee (PNM-RESC), King’s College London (ref: 15/16-2320).

### Analysis

Data from both studies were analysed together, using reflexive thematic analysis (Braun & Clarke, 2022). Initially, a process of familiarisation with the data took place through transcribing audio recordings and reading the dataset as a whole. A first coding round was then used to identify the different concepts and information contained in the data, with a view to broadly organising the data. A second coding round then went through the data more closely, with a particular focus on identifying nuances and gaining an in-depth understanding of each topic contained in the data. Through this second coding round, themes began to be formed based on concepts that repeatedly came up across the data, and concepts that invoked debate and differences of opinion between focus group participants. Themes were then formed by grouping coding categories together according to an over-arching concept or principle captured by a group, and then checking back through the raw coded data to see if the data made cohesive sense under the theme. The final themes represent core values for qualitative diary digital research, each value covering an aspect of qualitative diary research that participants identified as important for them.

Researchers kept reflexive diaries throughout the research process, to support paying consistent attention to acknowledging and reducing the impact of their own experiences and assumptions on the study findings. Reflexive discussions took place following focus groups, and at all stages of the study planning and analysis, to support researcher reflexivity in discussing alternate perspectives.

For this study, we took a relativist ontological position with a social constructionist perspective[REF THIS]. We took this approach as we wanted to look at the experience of taking part in a qualitative diary study from a personal point of view, while also considering the social constructs at play when relaying personal experiences to a researcher audience. Mindful of the fact that data in a qualitative diary study depends on how participants express themselves, and feel able to express themselves, we were therefore primarily interested in how participants talked about their experiences, and in how they talked about expressing their experiences through their diary entries, and therefore felt a social constructionist perspective was best suited to this.

### Findings

The objective of this study was to understand the perspectives of two different participant groups on their experiences of taking part in qualitative digital diary research, and to explore what can be learned from this in terms of participants’ core values for qualitative digital diary research. The six themes we developed each cover a core value that participants identified as important for them in taking part in qualitative diary research: self-expression, flexibility, non-judgement, open communication, helpful reflection, and meaningful impact. Together, these themes highlight the ways in which participants can be supported to take part in qualitative diary research in ways that enabale them to make meaningful contributions. Participant quotes are identified according to each study, with recovery diary participants labelled “RY” and REACH participants labelled “RH”.

### Self-expression

This theme highlights the importance of supporting participants’ individual preferences for self-expression in their diaries. Participants valued the individuality of diary-keeping and felt that being able to engage in this in ways that best suited them in the moment was a key positive of digital diary methods. Participants in both studies had different formats available to use - including written, audio, and video recording - and found different benefits to each method, with choice of method generally reflecting what participants wanted to get out of each diary entry.

Written diary entries suited participants when they needed to think through what was on their mind in order to articulate it: *“I’d have to sort of think in my head like ‘what did I experience throughout like these two days?’ and then I’d like write it down slowly. But maybe that’s a blessing in disguise, because if I did voice recordings then it would be from here, to there [all over the place].” (RY2)* For others, writing was easier than trying to verbalise thoughts: *“typing is just the easiest because I find it hard to put everything into words when I’m speaking. It’s easier to just type it out.” (RH22)*. Writing also helped with privacy concerns, as diaries could be written anywhere privately without fear of being overheard, or of waking sleeping family members.

However, there were times when writing felt challenging, sometimes due to the physical act of typing on a phone, “*it can be hard to type out as much, you have to keep it a bit shorter.” (RY6)*. Communicating difficult experiences in writing was harder for some participants: *“if something particularly difficult happened, or it’s been a bit of an up and down day, I find that a bit easier to give an explanation in kind of, in a voice recording rather, it’s sometimes harder to get across in writing, I think.” (RY3)*.

Audio recording provided a different avenue for reflection and expression compared to writing. “*I found that it was, I really liked the voice to text option. So for me, it was easier to have more stream of consciousness, just to be able to say it, and also put it as I was thinking it through, rather than thinking and pausing and deciding how to write it down.” (RY9)*

Audio recording diary entries was generally preferred when participants wanted to use the diary as an outlet: “*I guess I really like it [audio recording] more when I just kind of like talk and like whatever comes out of my mouth comes out of my mouth, that’s how I’m feeling. Whereas if it was writing, I have to consider it a bit more” (RH29)*.

Video recording was an additional option for REACH participants. Many participants did not use this, referencing that being on camera was “*not my thing*” (RH18), so they used alternative methods - *“I would always choose the voice one because I was very shy if I want to do a video” (RH12)*. However, some participants did choose videos, citing ease of recording a lot of information in one go. One participant talks about using it as like their own personal Youtube channel, allowing them to reflect on their day to an imaginary audience of peers, which may have supported them to reflect and report on what they felt is most relevant to record: *“Some of my videos I’ve been acting like as if I have a personal YouTube channel. I don’t know why. But yeah.” (RH5)*

This theme highlights that having different options available made participants feel that they could contribute to the study even when they did not feel like talking/writing/recording, and that participants critically reflected on the benefits and challenges of different methods while they were taking part in the study. Participants reported that people may not know ahead of time what is going to be the most effective way to communicate their experience, so the different options ensured that there would be some way that worked for them.

### Flexibility

This theme highlights the importance of flexibility in supporting participant engagement. Participants reported that different schedules and options for diary entries make it easier to participate, as well as making the data provided more rich and valuable. Several participants reflected on the importance of capturing their in-the-moment experiences, rather than reporting on experiences at the end of the day:

*“Being able to record at multiple times through the day, as something happened, or what you found difficult at that time to catch it at that time, I think would be a good option…. especially in eating disorders thoughts, because later in the day you probably would have calmed down and it’s almost like you try to think of the emotions, just being able to literally just capture it on your phone as it’s happened, I feel would be a lot better.” (RY3)*

This quote illustrates how different types of recordings or diary entries capture different perceptions of experiences, and that the flexibility to do both in-the-moment and end-of-day reflections can be valuable. Similarly, some participants reported that squeezing in a diary entry at the end of the day meant that they had less time for reflection than they might have if they did earlier in the day during a free moment.

Participants valued the flexibility that the mobile app platforms gave them to take part, appreciating the multiple format options for entries, as mentioned in the previous theme, as well as the fact that it was on their phones which made it fit easily into their lives:

*“Most of the time we’re on our phones, you know, sending voice notes or typing paragraphs to our friends… So it just felt like that, like, it felt like another form of social media, like, literally just, you know, just talk to you.” (RH10)*

Similarly, reminder notifications from the apps were appreciated, supporting involvement and reducing burden of guilt when entries were forgotten, and allowing the diaries to fit into participants’ busy lives: “*I found that really good because sometimes I would just get caught up in revising and forget to do it, so it was very helpful to be able to have a reminder*.” *(RH2)*

Flexibility within the diary content was also mentioned: *“I think if there wasn’t a time thing [limit], then I would see it as more, like, flexible. Because I think the five minutes made me think, ‘Oh, it has to be five minutes long, so I need to set aside five minutes to do it.’ And then I’ll be, like, conscious, like, ‘Oh, I didn’t go into detail’” (RH17)*, highlighting the need for flexible entries to reduce potential anxiety or stress around planning entries. Some participants referenced keeping their own personal diaries as well, meaning that the diary entry they recorded for the study was less personal as they did not want to repeat things. Therefore, awareness of how the diaries for research fit into the context of the participants’ lives is important in fully understanding diary entries.

Participants suggested that getting into a routine of taking part would help keeping the diaries fit more easily into their lives. “*I think that a little bit of a long time would definitely have been helpful… getting into the habit of it, because they say it takes like 21 times of doing something before it becomes a habit. So a little bit longer would definitely have been helpful to cement into my routine.” (RY4)*. Different participants had different suggestions as to what would be a good habit-forming routine for them; some preferred daily entries for a shorter period of time, and some less frequent entries for longer. This emphasises the individuality of this and the importance of consulting with each participant and being flexible in approach to diary schedules.

However, taking ownership over involvement in the study was also felt to be important: *“I feel like because I volunteered to this study, so I sometimes feel I may forget to do some homework. If I am volunteering to do such things I think I also have some responsibility for that.” (RY5)*

Overall, flexibility was considered an essential part of a diary study that supported participants to take ownership of their involvement, and meant they could take part in ways that best suited them and made sense to them in the context of their experiences. Participants understood the value of flexibility for supporting individual involvement and control over data, and the value of this for the research as a whole.

### Non-judgement

The central concept of this theme is that participants need to feel the audience to their diary entries is non-judgemental and understanding, and that this is an essential part of their willingness to engage openly with a qualitative diary study. Key to this with the Recovery Diary Study participants was involvement of lived experience researchers:

“*I would want someone that has, I don’t know, been touched or knows about eating disorder recovery and everything that goes along with it. I think I would feel more comfortable because I think I’d feel less kind of alien-like towards them? That they’re not kind of looking at me like… like they’re thinking ‘what the heck is she on about?’ (RY7)*

This comment suggests that involvement of lived experience researchers would help break down researcher and participant power differences, with the researcher observing from the inside rather than looking in from the outside, helping people feel less judged and more able to freely communicate. A shared position of understanding and empathy would also facilitate communication and understanding:

*“If it’s somebody with lived experience you might feel less judged in a way, because they’ve gone through, not the same thing, but something of their own. And they kind of may relate to it. Somebody without lived experience may not understand what you’ve written… They might be like, that makes no sense to me. Whereas somebody with would go, ‘ok, yeah, I know what that is’.” (RY8)*.

Shared language and experiences would mean participants felt less pressure to explain their thoughts, and thus record diary entries more freely: *“You might not feel like you have to kind of explain so much, why something was so difficult perhaps, because you know, the person reading it would already kind of have an idea why you might be finding it difficult.” (RY6)*. Similarly, participants considered a lived experience researcher to have benefits for analysis: *“It’s really hard for people that haven’t gone through something similar in general, in like mental health, to understand any like… minute nuances that some people might write.” (RY4)*.

Overall, having lived experience researcher involvement would support participants to feel more comfortable, less removed from the researchers, and better understood: “*there’s that already initial position of empathy that they can really like see what you’re going through.” (RY4)*. And: *“If he or she doesn’t know anything about it [from lived experience] the analysis seems very neutral, but without some essential component.” (RY5)*.

However, some participants reported concerns with lived experience researchers being more likely to pick up on things related to their own experience, introducing bias into the findings, and reflected that having both types of researcher involved in analysis would be their preference. Participants also reflected that non-lived experience researchers would also have their own set of biases.

*“Perhaps they can go internally into their own relatability about lived experience, but it might skew the data in either direction that you might not get from somebody who’s looking at it from more of a distant perspective. But, I think that’s the beauty of having research that has both [lived experience and non-lived experience researchers], right? That you get the pros of both of those.” (RY9)*. There is a sense here that there is more a fear of misinterpretation because of other people’s experiences biasing them. This relates to the importance of understanding individual experiences and how each person’s experience is unique.

Another key part of non-judgement raised by REACH participants was their long term involvement with the research team as part of the wider cohort study. For the REACH study, researchers maintained contact with participants and their schools for several years prior to the diary study, including six waves of data collection and an extensive engagement programme (Richards & Robotham, 2022), allowing trusting relationships to be established with the research team that meant participants felt safe to speak freely within their diary entries: “*it did feel quite nice because it was like place where I was kind of able to talk about like what went wrong that week and let out all my feelings with you. (RH33)*.

Additionally, feeling a reduction of individual scrutiny was one potential positive of studies with larger numbers of participants: *“I think the whole like judgemental part comes into it. Like that you’re under the microscope all of a sudden, whereas if there’s a lot of us together…” (RY7)*.

### Open communication

This theme covers the importance of communication between researchers and participants being as open as possible. This builds on the other themes as open communication can only be possible when people are able to express themselves how and when they need to, and to an understanding audience.

Diaries offered a place where participants could be authentically themselves, and as an outlet for their thoughts and feelings. For some, this was an opportunity they did not have outside of the diaries.

“*Being involved in the diary entries has really helped me just know how I feel, because sometimes I don’t really talk about my feelings so it’s nice to be able to like talk and just let everything out*.” *(RH2)*.

Participants reflected on wanting to be open and honest about their experiences, with an awareness that this would help the research. *“I felt that because I knew this was not going to be read by somebody that I was having a reciprocal relationship with, in a sense that it wasn’t a therapist, it wasn’t a dietician, that we were following up with. I felt like I was able to just give my honest opinion, because I knew it was for research. And so I knew in my mind, oh well an authentic answer, whatever it is, is better” (RY9)*.

Alongside wanting to be as open as possible, participants also reported awareness that they were writing for research rather than themselves, and the audience they had in mind while recording entries directly affected how they communicated, as highlighted in the previous theme, ‘Non-judgement’. Some adjusted their writing to ensure they would be understood by others.

*“There was a lot of translation work, because when I’m writing a diary entry for myself… it’s not like sentences that would make sense to anyone. So yeah, it was kind of like making it make sense, but other than that, um… it wasn’t really mellowed as such.” (RY8)*.

For some participants there was a sense of directly writing to someone: *“It like helps you guys give an understanding about what we are like feeling, and like it helps me like express how I’m feeling about what I’m going through right now.” (RH1)* This sense that someone was listening was also comforting: *“It felt nice to just let everything out, just typing everything, my emotions out, everything. Just because I personally like, it makes me feel nice, even if I talk to a complete stranger about a horrible day that I had, just having someone that I can tell or knowing that someone is just listening to me or like reading about it is nice, honestly, it’s really nice.”* (RH46)

Others used it more as an outlet, treating the diary as a space for personal reflection: “*I kind of felt like I was going to a therapist and kind of like talking about me all the time. I really liked it, I thought it was nice, I guess like getting those thoughts out into words”. (RH29)*

Participants in the REACH study more often referenced thinking of the researcher directly as their audience than those in the Recovery Diary Study: “*It like helps you guys give an understanding about what we are like feeling, and like it helps me like express how I’m feeling about what I’m going through right now. And like you guys are very like, since Year 7 like you guys have been very helpful to everyone, in my opinion, in my school.”(RH1)*. This may be due to the REACH participants having participated in baseline interviews with the researchers, and being involved in the wider REACH project for several years.

The prompts and questions that participants responded to in their diaries could either help or hinder open communication. Some prompts framed experiences and made participants feel they could not report on other things they felt were valuable, or the very question shaped how they thought about what to write in a constraining way (e.g. the “thoughts or feelings” rather than “experiences” which is more open). *“If I had to answer [specific] questions, I might have gotten too analytical about it, but this let me tap into a bit more of a holistic like approach to it I think.” (RY9)*.

However, some felt that diary prompts restricted open communication: “*I remember being slightly frustrated from them [the prompts], a little bit. For me I would have rather wrote just kind of freely, in just like a reflective journal for the day. But that’s my, probably my own personal style of writing, sort of thing.” (RY3)*. Others reflected on questions not seeming quite right for the circumstances at times: “*the questions were kind of similar which was a bit hard, because if I was doing it I don’t know when not much had happened it was quite weird” (RH8)*. This emphasises again the importance of supporting individual differences and ways of expression, and of flexibility with prompts around different circumstances and timeframes.

Concerns around openness of communication were raised around risk and safeguarding discussions, where participants were aware that words recorded in moments of heightened emotions in a diary entry might lead to more worrying than necessary.

“*If I had been having a really bad day, if I knew the information would perhaps be used or be alerted to somebody else, I think I might be more hesitant about what to write down and how much depth to go into. So kind of actually asking the person who wrote the entry, are you ok, we just want to know whether you are still feeling this bad, or whether you would like us to kind of get further help for you?” (RY6)*. This was particularly important with in-the-moment entries being available, as theres is a difference between venting in the moment and actually having lasting bad thoughts at the end of the day.

### Helpful reflection

The key concept of this theme is that participants valued taking part in diary study as it provided a place for helpful reflection.

“*It was quite nice, to be honest, because it was the end of the day and I was in bed and I could finally just put all the feelings and everything, like what happened during the day, into a diary. I just thought of it as my notebook for expressing myself and expressing how I felt. It was good. It was like a reflecting type of thing.” (RH22)*.

Diaries enabled participants to think through what was going on in their lives, raising awareness of how they were feeling, and awareness of day-to-day changes and coping strategies: “*it brought up things that I kind of wouldn’t have thought about like otherwise. Um… which was actually quite helpful, just to notice like how things differ day to day, and kind of what general strategies I use, even if they’re subconscious.” (RY8)*. This had a positive impact on participants: “*I think the diary itself was pretty good for my mental health because I don’t talk – I wouldn’t talk about stuff kind of like if I’m upset or something’s going on… so I think kind of having the diary itself kind of helped just with your kind of mental health. (RH39)* This may have been especially important for the participants in the REACH study, as they were keeping diaries during the time of Covid-19 related lockdowns and social restrictions.

Diaries supported participants to structure their thinking and recognise emotions: “ *I think it just made me like think and realise, “Oh whoa, this is how I felt”. So… this is how, yeah, this is how I felt. Like sometimes we’re not recognising some of the emotions that I feel. Like it helped me like… it’s kind of relaxing doing the diary entry and writing down everything that I felt.” (RH41). “It kind of helps you identify your own triggers as well.” (RY8)*.

Participants also considered the potential negatives of the type of reflection required by taking part in a qualitative diary study. Raising awareness of difficulties could be tricky as it emphasised to them that things needed to be worked on: “*I think that it highlighted, unknowingly some things that are still difficult in recovery. And it gave me a chance to practice some more of those things that I still want to keep working at… I think wasn’t necessarily a down side, but it was am unexpected difficulty of doing the app itself. The process of it, not necessarily the outcome of it.” (RY9)*. Some participants said it didn’t raise their awareness overall, as they only thought specifically about recovery when they actually did their diary entry.

Other participants acknowledged that it was sometimes difficult to actually write down the things going on in their heads: *“A lot of the time what I just want to put down is I ate a sandwich, it was good. Um, without the complexity of yes I ate a sandwich, after all the complexity of these like mental gymnastics to decide, because that is having to dig in for that information in my head, it’s a lot” (RY2)*.

Overall, there are ways to support positive and helpful reflection through taking part in a qualitative diary study, and researchers must be aware of potential negatives of reflecting on difficulties and have plans for how to support participants with this.

### Meaningful impact

This theme highlights the ways participants want to have control over their data and what happens with it. Participants were motivated to help others by taking part in research and emphasised the importance of diaries in helping improve understanding of their individual experiences. They highlighted the importance of understanding individual experiences, and factors in their lives that impact on these, and that this increased understanding could lead to more tailored mental health support. All participants highlighted the value of collecting diary data: *“Especially if we’re trying to inform like individualised recovery, rather than recovery with a set idea of what it should look like for everyone.”* (RY2)

Capturing and understanding the individuality of experiences was of major importance to participants, which can be understood in the context of one participant’s concern around diary entries potentially being reduced to generic points or stereotypes: *“[I would think] what was the whole point of my pouring my heart out, [if]I thought I was doing something that would help people and as it turns out, no, just getting general things*.” (RY1)

Within the REACH study, participants frequently referenced how they felt the importance of relating their experiences, helping the cause of mental health research in general, and helping others understand what people their age were going through.

“*I liked to just, you know, just documenting what’s happened, what I’d been feeling. And, yeah, if it goes towards a good cause helping with mental health, then that’s a good thing. So I was always happy doing it*.” (RH32). Another participant was one of several who mentioned the dual benefits to themselves and the research of keeping their audio diary: *“Saying stuff out loud is great, and just being able to help, because I know this kind of stuff can help” (RH4)*.

Alongside the lived experience involvement in research highlighted above, which participants felt would benefit the analysis and study findings, one participant highlighted the value of participant involvement in the diary analysis: “*I think also, keeping a diary but then having the participant’s own mini kind of analysis of their own diary, to pick out what they feel is important to them. Because then it might highlight and correspond with other diary entries for themes, that might not necessarily be what’s necessarily important to me*.” *(RY3)*.

Within the recovery diary study focus groups, we discussed the possibility of diaries being used in large dataset analyses such as big data studies. Participants talked about how this different use of diaries would affect how they completed their diary entries - such as ‘*rambling more*’ *(RY4)* and worrying less about specifically writing for someone else to understand. However, another participant reported the opposite, saying they might write less if no one was going to bother reading it: “*It’s almost like what’s the point in writing a diary entry, which is meant to be personal, which is meant to be looked at more personally, just for it to be churned through a computer, to be turned into stats*.” *(RY3)*. This shows important things about potential differences in characteristics of participants who might be interested to take part in either type of study. Concerns around privacy and data sharing were also discussed by participants, who reflected that this might be more of an issue with a large database of diaries than with smaller scale research.

This theme highlights the fact that participants have a strong focus on the role that understanding their individual experiences will take in the research, and the benefits this could have for supporting those with mental health difficulties in the future.

## Discussion

In this paper we sought to explore participants’ experiences of taking part in a qualitative digital diary study, and to provide a framework for centring participant values and preferences in future qualitative diary research. Our findings highlight six essential values: self-expression, flexibility, non-judgement, open communication, helpful reflection, and meaningful impact. Together, these themes highlight the ways in which qualitative digital diary research can support participants to take part in research in ways that enabale them to make meaningful contributions. With a focus on each of these values as a central requirement, qualitative diary methods can offer a collaborative approach where participants are in control of their own contribution and benefit from it. Participants were enthusiastic about keeping diaries for research, stressing the importance of qualitative diaries as a way to understand individual experiences and journeys, and thought that this would be extremely valuable in informing mental health understanding and interventions.

There are some points worth emphasising from the findings. Firstly, participants’ experiences of taking part were largely positive, but, as reported in the ‘helpful reflection’ theme, at times some found that reflecting on things in their diary entries made them feel sad, or raised awareness of things they needed to address. This is a natural part of diary keeping in any form, but when it is for research, it must be made clear at the outset of the study that this could be a possibility, and how the research team will provide support with managing this during the study if necessary, so that participants can make an informed decision about taking part. It is worth noting that even when participants in our studies talked about diaries making them feel sad, they still noted that it was worthwhile and they were glad they had this space for reflection.

The second point is about consideration of audience. Participants generally had a clear idea in mind of who is reviewing their diaries, and that this directly impacted on how and what they communicated. It is therefore essential for the audience to be clear to the participants at the outset, requiring the researcher to be authentic and to reflect on their identity and how this relates to their participants. A consequence of this awareness of audience is that diaries do not represent a straightforward ‘window’ into participants’ worlds (as suggested by Bolger et al. (2003)), and that while researcher influence may be reduced in comparison to other methods, the researcher is still present to an extent as a co-constructor of the diary entries and interpretations. Participants valued having lived experience researchers involved in diary analysis, which should be particularly prioritised in qualitative diary studies due to the personal and private nature of this type of research compared to interviews and other methods.

This work builds on research exploring the utility and acceptability of qualitative diary methods in other fields, such as work on audio diaries in work psychology research. Crozier and Cassell (2016) found audio diaries support participants to engage in longitudinal research in a less burdensome way, and our findings expand on this by eliciting further ways to support engagement with and benefits for participants in qualitative diary studies. Williamson et al. (2015) reviewed some ethical and methodological issues with the use of audio diaries, including around prompts and communication of risks within participant diaries, which our study has provided participant reflections on to support use of this method within mental health research. Our findings also provide some suggestions as to how to reduce the conflicts between researchers’ and participants’ understanding of diaries reported by Seguin et al. (2022), by emphasising flexibility, open communication and shared understanding. Finally, the participant reflections presented here address some assumptoms acknowledged by Cudjoe et al. (2022) that by using diaries, researchers provide participants with enough time to reflectively complete research. Our findings show that this is very much dependent on the how centred in participants lives and preferences the study design is.

There are some limitations to consider with this study. We used data from two very different groups of participants, and did not carry out sub-group analyses, and so the nuances of each group’s experiences and preferences for diary studies may not be fully captured in the findings. A further limitation is that the diary experiences questions were only a small part of the data collected for the REACH study, so some topics were unable to be fully explored with this participant group. Additionally, not all participants who took part in the diary keeping phases provided their perspectives on taking part, so it is possible that those who have not had their voice heard may have different persepectives on diary studies.

However, the diversity of participant groups is also a strength in that experiences, preferences, and concerns that were relevant to both participant groups were found, which provides an excellent starting point for those considering how best to support participants in their own qualitative diary studies. The participant populations in both groups were diverse in terms of ethnicity and mental health experiences, giving a broad understanding of participant experiences across perspectives.

## Conclusions

Participants in this study were very positive about qualitative diary methods, and particularly on the focus that they can give research in terms of understanding individual experience and meaning. This exploration of participant values for taking part in qualitative diary research offers a starting point for researchers considering using this method to consider how best to support and involve participants in their research, beyond simply as subjects providing data.

## Figures and Tables

**Table 1:** Participant characteristics, Eating Disorder Recovery Diary Study

**Age (years)**	20-29 – 6 30-39 – 1 40-49 – 2
**Gender**	M – 2 F – 6 Prefer not to say – 1
**Ethnicity**	White British – 5 Asian/Asian British – 3 Mixed/multiple ethnic groups – 1
**Highest education** **level**	Bachelor’s degree – 7 Masters or other post-graduate degree – 2

**Table 2:** Participant characteristics, REACH Diary Study

**Gender**	Girl – 24
	Boy - 16
**Ethnicity**	Black African - 17 Mixed - 7 White British - 4 India, Pakistan, Bangladesh - 3 Black Caribbean – 2 Latino - 2 Arab - 2 Other White - 1 Chinese - 1 Other Asian - 1
**School year**	Y10 – 1
	Y11 – 14
	Y12 – 16
	Y13 – 9
**Free school meals**	Yes – 20
	No – 20
**At risk of mental health difficulties**	Yes – 15
	No - 25

Notes: School year typical ages: Y10, age 14-15; Y11, 15-16; Y12, 16-17; Y13, 17-18. Free school meals status is a proxy for socioeconomic status; at risk of mental health difficulties is indicated by a ‘high’ score on the Strengths and Difficulties Questsionnaire.
